# Time estimation and passage of time judgment predict eating behaviors during COVID-19 lockdown

**DOI:** 10.3389/fpsyg.2022.961092

**Published:** 2022-08-22

**Authors:** Eve A. Isham, Sara Lomayesva, Jiaxuan Teng

**Affiliations:** Consciousness-Action-Time Lab, Department of Psychology, University of Arizona, Tucson, AZ, United States

**Keywords:** snacking, overeating, eating behaviors, time perception, passage of time judgment

## Abstract

Poor eating habits often lead to health concerns. While mental health conditions such as stress and anxiety have been linked as predictors for eating behaviors, cognitive factors may also contribute to eating practices during the early stages of the mandatory COVID-19 lockdown. In the current study, participants responded to a survey that asked them to judge the passing of time (PoTJ) and to produce short intervals (*via* a time production task) as an index of the internal clock speed. Additionally, they responded to questions about snacking frequency and the tendency to overeat during lockdown. We observed that those who judged time to pass slowly also reported a greater tendency to snack and overeat during the pandemic. Additional analysis also revealed that the effect of PoTJ on snacking is moderated by the internal clock speed such that those who felt time was passing by slowly, and in combination with a faster internal clock (as indexed by shorter duration production), had a greater tendency to snack. The results suggest that different aspects of temporal cognition play potential roles in influencing different types of eating behaviors. Our findings therefore have implications for eating disorders, along with the potential of time-based intervention or behavioral modification approaches.

## Introduction

The emergence of the COVID-19 pandemic spurred orders for quarantine across the United States in March 2020. People were told to stay home from work and refrain from in-person social settings to protect themselves and others from the spread of the virus. The absence of regular social activities resulted in more time for individuals to spend in isolation. This sudden interruption led to a number of changes in people's daily routines, behaviors, and psychological experiences including changes in eating behaviors (e.g., Miniati et al., 2021; Borisenkov et al., 2022). Often, disordered eating behaviors especially during distress are examined in the contexts of mental health (Buckland et al., 2021). During the pandemic social isolation, uncertainty, and fear of contracting the virus resulted in feelings of depression and anxiety in many individuals (Marazziti et al., 2020; Poli et al., 2020), and this has been linked to an increase in poor eating habits during quarantine, including emotional overeating (Gao et al., 2022), and consumption of unhealthy food (Landaeta-Diaz et al., 2021; Salazar-Fernandez et al., 2021). In addition, past studies have linked eating disorders to anxiety and depression (Godart et al., 2002; Keel et al., 2005; Swinbourne and Touyz, 2007; Swinbourne et al., 2012; Garcia et al., 2020; Sander et al., 2021) and significant changes in appetite and eating habits are established diagnostic criteria for depressive and anxiety disorders (American Psychiatric Association, 2013). Anxiety, in particular, has been linked to overeating or binge-eating (Polivy et al., 1994), and emotional eating in obese individuals (Schneider et al., 2010).

While mental health and eating behaviors have been linked, we asked whether other factors may also be involved. For instance, in addition to changes in eating behaviors, individuals in lockdown also reported a distortion in temporal experience in which time appeared to progress at an abnormal rate (Droit-Volet et al., 2020; Ogden, 2020; Martinelli et al., 2021), prompting the question of whether temporal experience and how we represent time also play a role in our eating behaviors during the pandemic. Past studies have suggested there is a link between time perception and eating behaviors, reflecting the connection between temporal cognition (e.g., interval timing and time passage judgment) to temporal discounting, reward sensitivity (Vicario and Felmingham, 2018), and cognitive control (e.g., Dietrich et al., 2014). Temporal discounting refers to a decision behavior involving tradeoffs between reward values that vary at different points in time. A greater degree of temporal discounting is observed when an individual chooses a lower value reward instead of higher value reward because the former is delivered sooner than the latter. A theoretical perspective posits that the degree of temporal discounting is associated with an individual's perception of time intervals. Perceiving time as short might motivate a person to wait for a larger reward (and therefore skip a small immediate reward) whereas perceiving time as longer might motivate a person to choose an immediately available but smaller reward. As such, one might predict that if the between-meal intervals seem shorter than they are, then the consumption of the next meal might be prolonged. On the other hand, if the between-meal intervals seem longer, the consumption of the next meal would be sooner.

Empirically, it has been shown that those who estimate time to be longer are also more responsive to immediate rewards rather than delayed rewards (Kim and Zauberman, 2009; Baumann and Odum, 2012; Suo et al., 2014). Relevant to eating behaviors, Rodin (1975) observed that when bored, overweight individuals perceived time as passing more slowly than individuals of standard weight; subsequently the overweight individuals would eat sooner. On the other hand, when not bored, they would judge time to pass quickly and would wait to consume food at a later time, suggesting a connection between boredom, passage of time judgment, and eating behaviors. In addition, work by Vicario and Felmingham (2018) examined dysfunctional time estimation in anorexia nervosa's eating behaviors. Generally, anorexia nervosa is a psychiatric disorder that is characterized by severe eating restriction and excessive self-control (Vicario and Felmingham, 2018). Previously, anorexia nervosa has been shown to have reduced temporal discounting behavior, displaying a greater ability to ignore smaller immediate rewards (e.g., food) in exchange for larger delayed rewards (e.g., weight loss). Vicario and Felmingham observed that anorexia nervosa had a greater tendency to underestimate intervals compared to control, supporting the hypothesis that such tendency to judge between-meal intervals as shorter likely contributes to anorexia nervosa's ability to prolong food consumption.

Physiological evidence in support of the potential link between time perception and eating behaviors has also been documented. A recent finding demonstrated a connection between eating and cerebellar activity, which is also involved in timing and time perception. A subset of neurons in the anterior deep cerebellar nuclei are activated by food intake, and subsequently such activity terminates additional food intake by increasing striatal dopamine levels (Low et al., 2021). In separate research, an increase in striatal dopamine and heightened cerebellar activity have been linked to sub- and supraseconds interval timing (e.g., Ivry and Keele, 1989; Ivry and Hazeltine, 1995; Ohmae et al., 2017; Kunimatsu et al., 2018). Research on eating disorders has also demonstrated that individuals who consume large amounts of food display different activities in the dorsolateral prefrontal cortex (DLPFC), a brain network involved in the processing of time information (Lewis and Miall, 2006). Furthermore, abnormal activity in other time-related brain structures, namely the striatal-cortical network (Kullmann et al., 2012; Nummenmaa et al., 2012) and the insula (Critchley et al., 2004), have been observed in obese individuals (Watkins et al., 2016). Collectively, these previous studies have further examined the extent to which time perception and eating behaviors are related, potentially paving the way for temporal-based interventions for eating behavior modification.

Given prior evidence suggesting an overlap in mechanisms related to time perception and eating behaviors, along with preliminary reports of abnormalities in both eating and time perception as a result of disruptions to daily life, the current study investigated how temporal cognition, along with mental health, predicts a change in eating behaviors during COVID-19 pandemic lockdown. Specifically, we examined how subjective experience of time passage and objective duration estimation performance predict overeating and snacking habits during lockdown. Time perception research has categorized temporal experiences according to their theorized origins. The experience of time passage (as indexed by the passage of time judgment; PoTJ) is considered a subjective interpretation that results from environmental contexts or health status (see Droit-Volet et al., 2013 for review). It is believed to reflect an introspective comparison between one's own internal time and the time of the world (Minkowski, 1968/1988), and this temporal experience was reported as highly distorted during the early stages of the pandemic lockdowns (Ogden, 2021). On the other hand, duration judgment or interval timing (achieved *via* a time production (TP) task) reflects a temporal experience that results from fundamental changes in the mechanisms involved in the representation of duration. For example, a cognitive model of time perception posits a pulsating internal clock as underlying duration estimation, and these pulses summating in the “accumulator,” leading to an interpretation of duration (Gibbon, 1977). Mental health or psychological status could alter the speed of pulsation, leading to an alteration in duration judgment. Different from interval timing, Droit-Volet and Wearden (2015) emphasized that additional influences are necessary to contribute to the judgment of time passing faster or slower, as the judgment simply cannot depend only on the number of pulses. Evidence suggesting the dissociation between PoTJ and duration judgement has been reported. For instance, when performing a PoTJ task along with a time production and time estimation task, no correlation between PoTJ and the two duration estimation tasks was found (Droit-Volet and Wearden, 2016). Moreover, patients with depression have reported time passing slowly but they perform similarly to control participants on duration judgment tasks (Thönes and Oberfeld, 2015).

Given the differences between PoTJ and duration estimation, we anticipated that they would contribute differently toward certain eating behaviors during the pandemic. We performed regression analyses to determine whether time passage experience (*via* PoTJ) and duration estimation *via* time production (TP) performance, along with depression, state, and trait anxiety level, predict eating behaviors such as snacking when bored and overeating during the pandemic lockdown. Often, the source of unhealthy eating practices is discussed in the context of mental health, physiological make-up, or socioeconomic status; all these factors, however, may have temporal cognition as a common root when exposed to extraordinary circumstances.

## Materials and methods

### Participants

A total of 160 volunteers were recruited to participate in online survey during the mandatory COVID-19 lockdown between April and May 2020. Of these, 137 participants (56 males, 92 females, and 1 unreported; mean age was 23.46 years old) had complete datasets and met the inclusion criteria (i.e., time production performance within two standard deviations). All participants had the option of receiving course credit or monetary compensation for their participation. All participants consented to the study protocol approved by the Institutional Review Board of the University of Arizona.

### Procedures

The survey was built and administered *via* the Qualtrics platform (Qualtrics, Provo, UT). Within the survey, a time production task, along with questionnaire regarding their perception of time passage and eating behaviors were assessed (see registered OSF https://osf.io/q3ryn for details). Participants were instructed to complete the survey without external distractions and without looking at a clock.

#### Time production task

The time production task consisted of 10 trials distributed throughout the survey. For each trial, the participant was instructed to produce a duration of 1 min by pressing a key on the computer keyboard twice; the first press indicated the onset of the duration, and the second press indicated the offset of the duration. The produced time for each trial was captured by the Qualtrics survey through a “Timing” question. The duration between the onset and offset presses was then used to calculate the ratio index of time perception. Ratio refers to the quotient between the produced duration and actual duration. A ratio of 1 indicates that the produced duration is the same as the actual duration. A ratio greater than or less than 1 indicates that the duration is over or under produced, respectively. Datasets with a time production ratio outside of the two standard deviations were excluded from the analyses.

#### Passage of time judgement and eating behaviors

As part of the survey, the statement “I feel time is going by slowly” was administered to capture the subjective experience of time passage and disorientation. A Likert scale (1 = not at all, 5 = all the time) was used to report the frequency in which the participants had experienced time passing by slowly BEFORE and DURING the mandatory distancing orders.

As part of the same survey, the statement “I tend to eat snacks when bored” and “I tend to overeat” were administered to assess for eating behaviors before and during the mandatory distancing periods. A Likert scale (1 = not at all, 5 = all the time) was used to report the frequency in snacking or overeating habits BEFORE and DURING the mandatory distancing orders. The order of BEFORE judgment always preceded the DURING judgment. Responses regarding the BEFORE period for all participants was a retrospective judgment. Responses regarding the DURING period reflected the present state as the data collection was concurrent with the COVID-19 mandatory distancing orders.

#### Depression and anxiety measures

Participants completed the Depression Anxiety Stress Scales (DASS; Lovibond and Lovibond, 1995) and the State-Trait Anxiety Inventory (STAI; Spielberger et al., 1983). The DASS measures depression through several constructs reflecting DSM-5 depression symptomology, as well as characteristic signs of arousal and stress. The STAI has two forms that measure two main types of anxiety: state (STAI-S) and trait anxiety (STAI-T).

#### Data analyses

All statistical analyses were conducted in IBM SPSS Statistics 28.0. Changes in snacking and overeating behaviors (i.e., rating difference; described below) were subjected to separate regression models. PoTJ, time production, and mental health measures served as predictors in these models. Subsequently, interaction effects in the regression analyses were further examined in a secondary between-subjects ANOVA.

##### Rating difference

The rating difference indexed the change in one's perception of time passage (PoTJ) or behaviors (snacking and overeating) as they entered the pandemic lockdown. The rating difference was calculated by subtracting the rating value experienced during and before the pandemic lockdown (DURING rating—BEFORE rating), resulting in nine possible difference values ranging between −4 and 4. We then converted these difference values to positive values ranging between 1 and 9. For PoTJ, a recoded value of 1 indicates that time was passing much faster, and a recoded value of 9 indicates that time was passing much slower during quarantine. For snacking and overeating tendency, a recoded value of 1 indicates a much lower tendency to snack or overeat, and a recoded value of 9 indicates a much higher tendency to snack or overeat during quarantine in comparison to before quarantine.

## Results

### Predicting the tendency to snack when bored

Of the participants, 137 completed the questionnaire related to snacking. We observed that 54 participants (39.4%) felt a greater tendency to snack during quarantine and 21 participants (15.3%) reported a lower tendency to do so. The remaining 62 participants (47.4%) reported no difference in their snacking behavior. The rating difference was subjected to three separate regression models that included depression, trait, or state anxiety as described below. As the primary predictors, PoTJ and time production were entered into all three models. In addition, each regression model also included a mental health score (depression, state anxiety, or trait anxiety) as a predictor.

#### PoTJ + time production + depression model

The regression model was significant, *F*(7,136) = 4.054, *p* < 0.001 with *R* = 0.425. The results revealed that PoTJ (*p* = 0.005) and the interaction between PoTJ and time production (*p* = 0.008) were the only significant predictors. Neither depression nor other interaction terms were significant predictors in the model (Table 1).

**Table 1:** Snacking regression coefficients for predictors passage of time judgment (PoTJ), time production (TP), and depression (DASS).

						**95% Conf Int for B**	
	**Unstandard. B**	**Coefficients Std. Error**	**Standardized Coeff Beta**	***t***	***p***	**Lower bound**	**Upper bound**
(Constant)	4.433	0.619		7.166	<0.001	3.209	5.657
PoTJ	0.198	0.07	0.238	2.828	0.005	0.059	0.337
TP	−0.368	0.333	−0.1	−1.104	0.272	−1.027	0.291
DASS	0.007	0.011	0.06	0.655	0.514	−0.014	0.029
PoTJ × TP	−0.695	0.259	−0.242	−2.68	0.008	−1.208	−0.182
TP × DASS	0.021	0.032	0.065	0.654	0.514	−0.042	0.083
PoTJ × DASS	0	0.007	−0.003	−0.031	0.975	−0.014	0.013
PoTJ × TP × DASS	0.002	0.021	0.01	0.099	0.921	−0.039	0.043

#### PoTJ + time production + trait anxiety model

The regression model was significant in predicting snacking frequency, *F*(7,136) = 4.154, *p* = 0.001 with *R* = 0.429. The regression results revealed that PoTJ (*p* = 0.005) and the interaction between PoTJ and time production (*p* = 0.019) were the only significant predictors. Neither trait anxiety nor other interaction terms were significant predictors in the model (Table 2).

**Table 2:** Snacking regression coefficients for predictors passage of time judgment (PoTJ), time production (TP), and trait anxiety (STAIT).

						**95% Conf Int for B**	
	**Unstandard. B**	**Coefficients Std. Error**	**Standardized Coeff Beta**	***t***	***p***	**Lower bound**	**Upper bound**
(Constant)	4.347	0.658		6.602	<0.001	3.044	5.649
PoTJ	0.207	0.072	0.243	2.867	0.005	0.064	0.351
TP	−0.382	0.32	−0.106	−1.196	0.234	−1.015	0.25
STAIT	0.005	0.009	0.05	0.6	0.549	−0.012	0.023
PoTJ × TP	−0.615	0.258	−0.213	−2.385	0.019	−1.126	−0.105
TP × STAIT	0.001	0.027	0.002	0.024	0.981	−0.052	0.054
PoTJ × STAIT	0.006	0.006	0.078	0.91	0.365	−0.007	0.018
PoTJ × TP × STAIT	−0.009	0.022	−0.038	−0.419	0.676	−0.052	0.034

#### PoTJ + time production + state anxiety model

The regression model was significant, *F*(7,136) = 4.366, *p* < 0.001 with *R* = 0.438. Similar to the Trait Anxiety and Depression models, the State Anxiety analysis revealed that PoTJ (*p* = 0.002) and the interaction between PoTJ and time production (*p* = 0.028) were the only significant predictors. Neither state anxiety nor other interaction terms were significant predictors in the model (Table 3).

**Table 3:** Snacking regression coefficients for predictors passage of time judgment (PoTJ), time production (TP), and state anxiety (STAIS).

						**95% Conf Int for B**	
	**Unstandard. B**	**Coefficients Std. Error**	**Standardized Coeff Beta**	***t***	***p***	**Lower bound**	**Upper bound**
(Constant)	4.556	0.642		7.101	<0.001	3.287	5.826
PoTJ	0.223	0.071	0.262	3.138	0.002	0.082	0.364
TP	−0.437	0.331	−0.121	−1.321	0.189	−1.092	0.218
STAIS	5.47E−05	0.008	0.001	0.007	0.994	−0.015	0.015
PoTJ × TP	−0.576	0.258	−0.199	−2.229	0.028	−1.086	−0.065
TP × STAIS	0.011	0.023	0.042	0.452	0.652	−0.036	0.057
PoTJ × STAIS	0.009	0.006	0.135	1.505	0.135	−0.003	0.02
PoTJ × TP × STAIS	−0.01	0.018	−0.055	−0.569	0.571	−0.047	0.026

#### Interaction between PoTJ and time production

In all three regression models, the passage of time judgment was a significant predictor in snacking, suggesting that those who experienced time passing at a slower speed also had a greater tendency to snack. In addition, the interaction between PoTJ and time production performance was consistently observed in all three models, suggesting an interaction between the two forms of temporal cognition. Mental health status did not appear to predict snacking frequency in the data.

**Figure 1:**
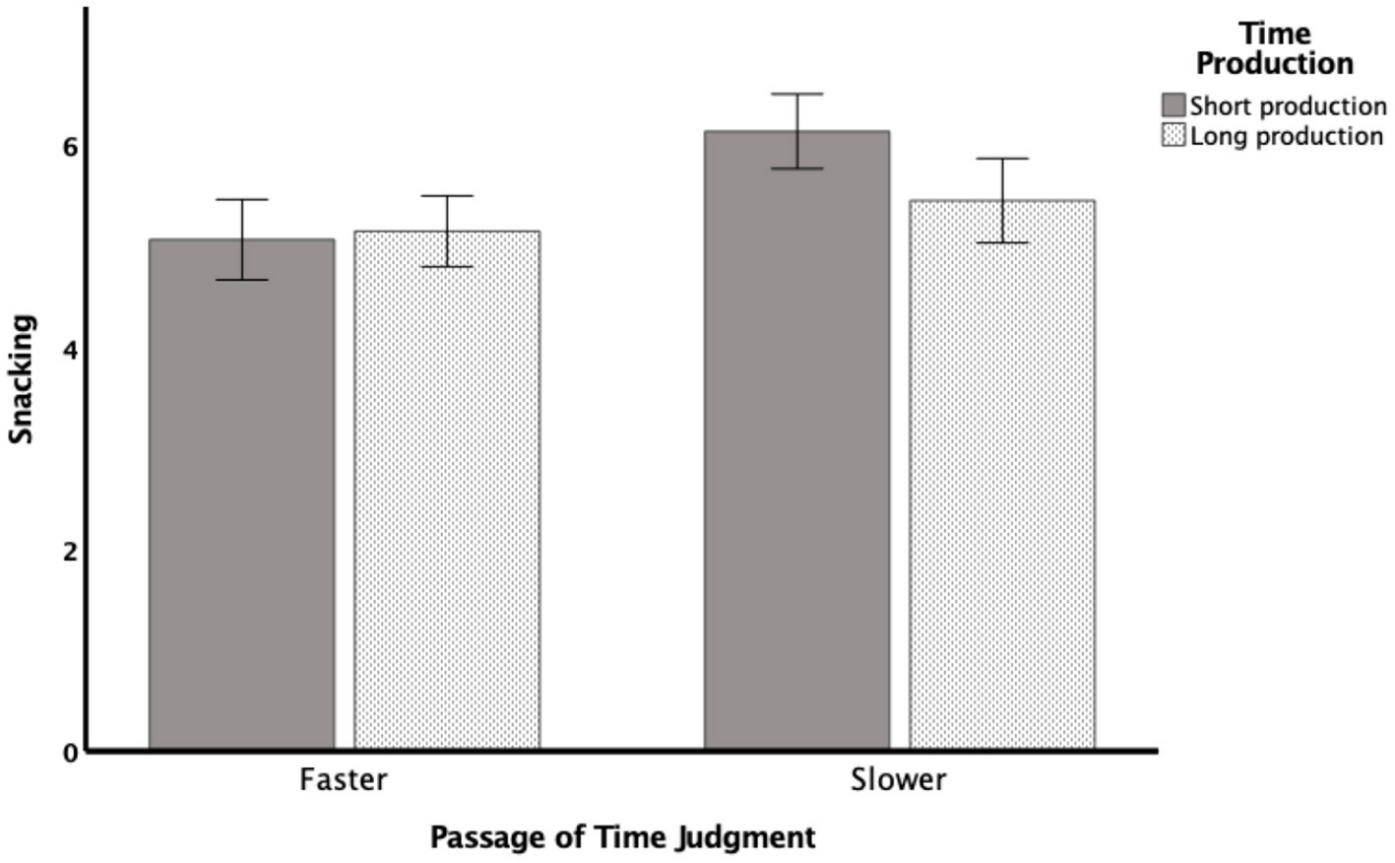
Rating difference in snacking behavior as a function of passage of time judgment and time production performance. There was a main effect of Passage of Time Judgment such that those who felt time was passing by at a slower rate also reported a greater tendency to snack (*p* < 0.001). In addition, there was an interaction effect (*p* = 0.05) such that among the participants who judged time to pass slowly, those who produced shorter duration also were more likely to snack than those who produced longer duration.

To better understand the interaction between PoTJ and time production, we categorized participants into two PoTJ groups (slower time passage and the same/faster time passage) and two time production performance groups (short-production and long-production) using the median split approach. Subsequently, we subjected the snacking rating difference to a 2 PoTJ (same/faster or slower) × 2 Time Production (short or long) between-subjects ANOVA. As shown in Figure 1 and in support of the regression results, the ANOVA revealed a main effect of PoTJ, *F* (1,137) = 12.281, *p* < 0.001, η^2^ = 0.085, such that those who judged time to be passing by slower reported a greater tendency to snack during the pandemic (*M* = 5.84, *SD* = 1.35) than those who judged time to pass by at the same rate or at a faster rate (*M* = 5.11, *SD* = 0.94).

Of interest, we also observed an interaction effect between PoTJ and Time Production, *F*(1,137) = 3.921, *p* = 0.050, η^2^ = 0.029. That is, we observed that for those who felt time was passing by slowly, the tendency to snack was further enhanced by production of a shorter duration (*M* = 6.14, *SD* = 1.36) than by production of a longer duration (*M* = 5.44, *SD* = 1.25), *t*(62) = 2.077, *p* = 0.021 (two-tailed). However, for those who felt time was passing by at the same or faster speed, there was no difference in the tendency to snack regardless of whether these individuals produced a shorter (*M* = 5.06, *SD* = 1.16) or a longer duration interval (*M* = 5.15, *SD* = 0.73), *t*(71) = 0.377, *p* = 0.707. Lastly, there was no main effect for Time Production, *F*(1,137) = 2.407, *p* = 0.123, = 0.018. That is, the short-production group (*M* = 5.64, *SD* = 1.37) did not differ significantly from the long-production group (*M* = 5.27, *SD* = 0.96) on the tendency to snack during quarantine.

### Predicting the tendency to overeat

Of the participants, 135 completed the questionnaire related to overeating. We observed that 52 participants (38.5%) felt a greater tendency to overeat during quarantine and 19 participants (13.92%) reported a lower tendency to do so. The remaining 64 participants (47.4%) reported no difference in their overeating behavior. The difference value in overeating was subjected to three separate regression models that included depression, trait, or state anxiety. The two primary predictors, PoTJ and time production, along with the interaction terms were entered into all three models. Regression results are highlighted below.

#### PoTJ + time production + depression

The regression model was significant, *F*(7,134) = 4.279, *p* < 0.001 with *R* = 0.437. The analysis revealed that PoTJ (*p* = 0.002) was a significant predictor, suggesting that the perceived slower passage was associated with an increase in overeating. The interaction between PoTJ and time production performance was also a significant predictor (*p* = 0.027). No other interaction terms were significant predictors in the model (Table 4).

**Table 4:** Overeating regression coefficients for predictors passage of time judgment (PoTJ), time production (TP), and depression (DASS).

						**95% Conf Int for B**	
	**Unstandard. B**	**Coefficients Std. Error**	**Standardized Coeff Beta**	***t***	***p***	**Lower bound**	**Upper bound**
(Constant)	3.918	0.607		6.451	<0.001	2.716	5.12
PoTJ	0.229	0.071	0.269	3.234	0.002	0.089	0.369
TP	−0.249	0.318	−0.07	−0.783	0.435	−0.879	0.381
DASS	0.019	0.011	0.156	1.716	0.089	−0.003	0.04
PoTJ × TP	−0.572	0.256	−0.199	−2.238	0.027	−1.077	−0.066
TP × DASS	0.001	0.031	0.004	0.044	0.965	−0.06	0.063
PoTJ × DASS	−0.008	0.007	−0.1	−1.149	0.253	−0.022	0.006
PoTJ × TP × DASS	0.013	0.021	0.058	0.613	0.541	−0.028	0.053

#### PoTJ + time production + trait anxiety

The regression model was significant, *F*(7,132) = 4.087, *p* < 0.001 with *R* = 0.432. The analysis revealed that PoTJ (*p* = 0.001) was a significant predictor. However, other predictors were not significant in the model (Table 5).

**Table 5:** Overeating regression coefficients for predictors passage of time judgment (PoTJ), time production (TP), and trait anxiety (STAIT).

						**95% Conf Int for B**	
	**Unstandard. B**	**Coefficients Std. Error**	**Standardized Coeff Beta**	***t***	***p***	**Lower bound**	**Upper bound**
(Constant)	3.874	0.669		5.794	<0.001	2.551	5.198
PoTJ	0.237	0.073	0.279	3.262	0.001	0.093	0.381
TP	−0.298	0.321	−0.083	−0.926	0.356	−0.934	0.338
STAIT	0.012	0.009	0.11	1.305	0.194	−0.006	0.03
PoTJ × TP	−0.456	0.259	−0.158	−1.757	0.081	−0.969	0.058
TP × STAIT	−0.01	0.027	−0.034	−0.38	0.705	−0.063	0.043
PoTJ × STAIT	0.005	0.006	0.074	0.855	0.394	−0.007	0.018
PoTJ × TP × STAIT	−0.016	0.022	−0.067	−0.727	0.469	−0.059	0.027

#### PoTJ + time production + state anxiety

The regression model was significant, *F(*7,134) = 4.536, *p* < 0.001 with *R* = 0.447. The regression results revealed that PoTJ (*p* < 0.001) and the interaction between PoTJ and state anxiety score (*p* = 0.046) were significant predictors. Neither time production performance nor other interaction terms were significant predictors in the model (Table 6).

**Table 6:** Overeating regression coefficients for predictors passage of time judgment (PoTJ), time production (TP), and state anxiety (STAIS).

						**95% Conf Int for B**	
	**Unstandard. B**	**Coefficients Std. Error**	**Standardized Coeff Beta**	***t***	***p***	**Lower bound**	**Upper bound**
(Constant)	4.133	0.645		6.407	<0.001	2.856	5.409
PoTJ	0.245	0.071	0.287	3.43	<0.001	0.103	0.386
TP	−0.346	0.331	−0.097	−1.044	0.298	−1.002	0.31
STAIS	0.006	0.008	0.066	0.788	0.432	−0.009	0.022
PoTJ × TP	−0.373	0.258	−0.129	−1.445	0.151	−0.884	0.138
TP × STAIS	0.004	0.023	0.015	0.165	0.869	−0.042	0.05
PoTJ × STAIS	0.012	0.006	0.181	2.013	0.046	0	0.023
PoTJ × TP × STAIS	−0.01	0.018	−0.051	−0.526	0.600	−0.046	0.027

#### Interaction between PoTJ and time production

**Figure 2:**
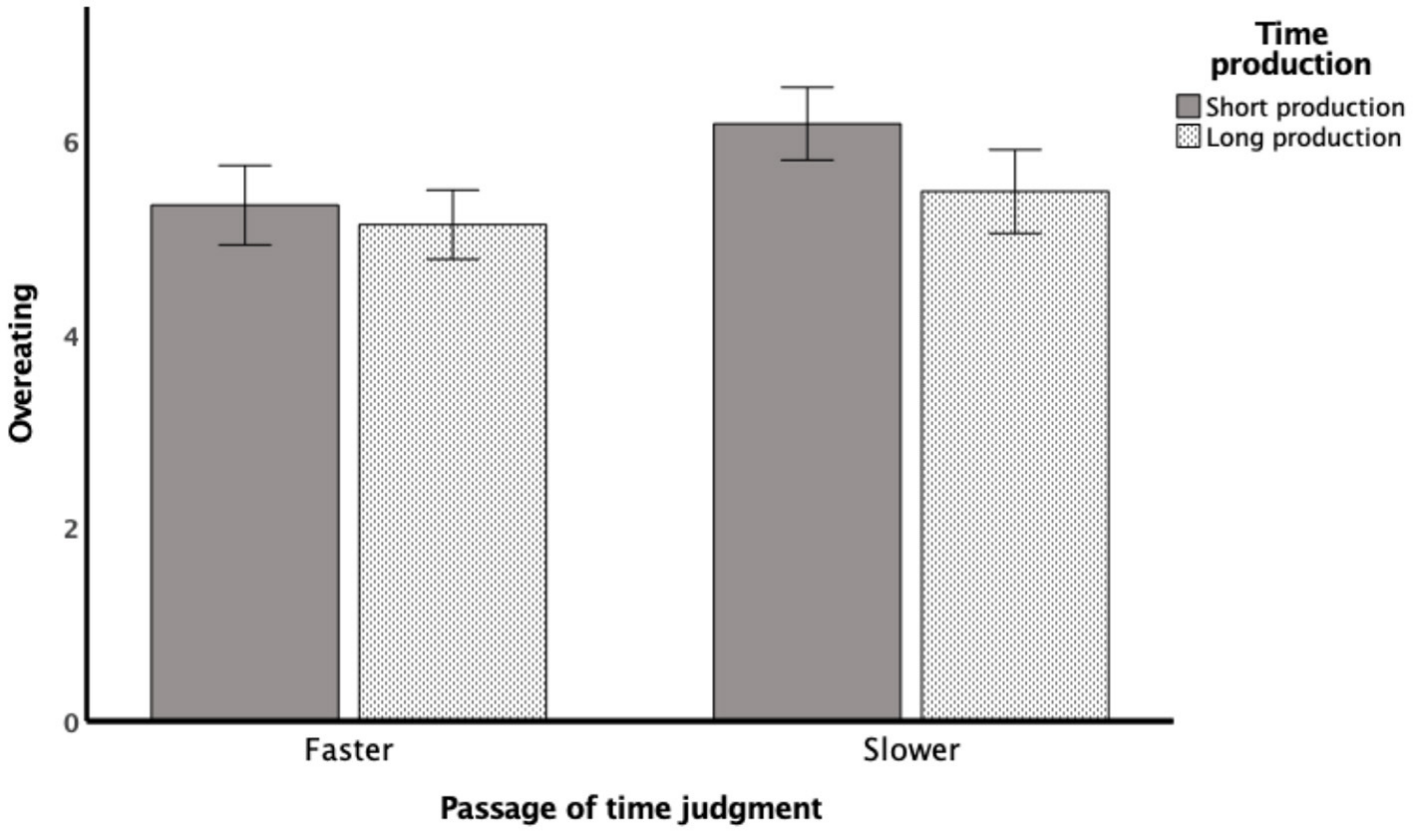
Rating difference in overeating behavior as a function of passage of time judgment and time production performance. There was a main effect of Passage of Time Judgment (*p* = 0.005) such that those who judged time to be passing by at a slower place reported a greater tendency to overeat during the pandemic than those who judged time to pass at a faster speed. In addition, there was a main effect of Time Production (*p* = 0.020) such that those who produced shorter durations reported a greater tendency to overeat than those who produced longer durations. There was no interaction effect between the two variables.

In all three regression models, the passage of time judgment was a significant predictor of overeating. However, depending on the model, the other predictors such as the interaction term between PoTJ and time production performance (as observed in the Depression model) and between PoTJ and state anxiety (as observed in the State Anxiety model) were also detected. To better understand the interaction between PoTJ and time production performance, the rating difference in the tendency to overeat was subjected to a 2 PoTJ (fast or slow) × 2 Time Production (short or long) between subjects ANOVA. As shown in Figure 2, there was a main effect of PoTJ, *F* (1,135) = 8.184, *p* = 0.005, η^2^ = 0.059, such that those who judged time to be passing by at a slower speed reported a greater tendency to overeat during the pandemic (*M* = 5.86, *SD* = 1.37) than those who judged time to pass at a faster speed (*M* = 5.21, *SD* = 0.95). In addition, though not observed in the regression results, there was a main effect of Time Production, *F*(1,135) = 5.554, *p* = 0.020, η^2^ = 0.020; the short-production group had a greater tendency to overeat (*M* = 5.64, *SD* = 1.37) than those in the long-production group (*M* = 5.26, *SD* = 0.971). However, inconsistent with the regression results, the ANOVA did not reveal an interaction effect between PoTJ and Time Production, *F*(1,135) = 1.823, *p* = 0.179, η^2^ = 0.014. The discrepancy between the regression and ANOVA suggests that the involvement of interval timing on overeating is unclear and may fluctuate depending on the presence of other factors (e.g., mental health).

#### Interaction between PoTJ and state anxiety

**Figure 3:**
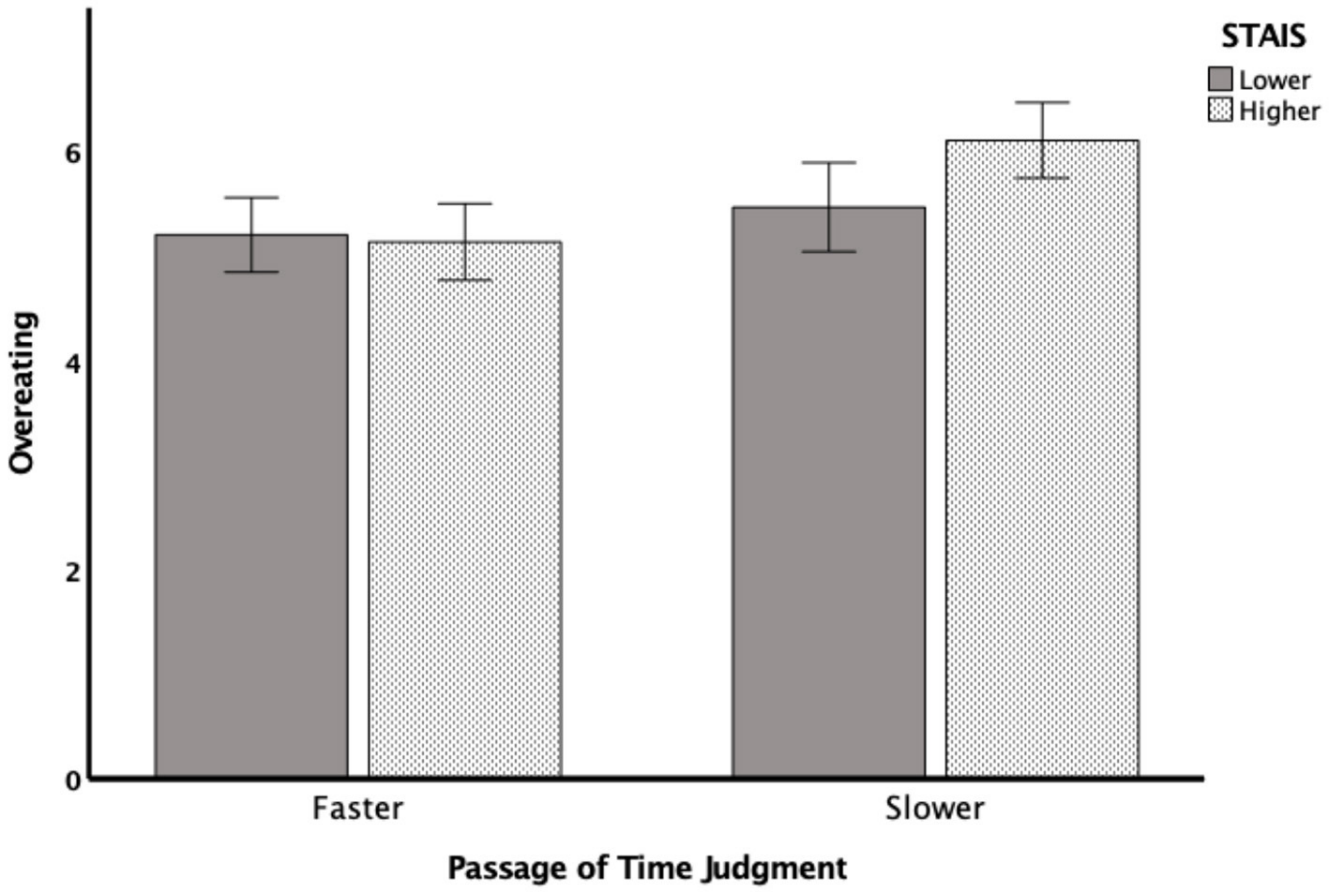
Rating difference in overeating behavior as a function of passage of time judgment and state anxiety score. There was a main effect of Passage of Time Judgment (*p* = 0.004) such that those who judged time to be passing by at a slower speed reported a greater tendency to overeat during the pandemic (*M* = 5.86, *SD* = 1.37) than those who judged time to pass at the same or a t a faster speed (*M* = 5.21, *SD* = 0.95). Moreover, there was an interaction between Passage of Time Judgment and State Anxiety (*p* = 0.043). The results suggest that among the participants who judged time as passing slower, those who experienced a greater degree of anxiety also reported a greater tendency to overeat than those who experienced a lesser degree of anxiety. However, anxiety did not play a role in those who judged time to pass at the same or at a faster speed. That is, overeating behavior did not differ among the low and high anxiety groups.

To further examine the interaction between PoTJ and state anxiety, we subjected the rating difference in the tendency to overeat to a 2 PoTJ (same/fast or slow) × 2 State Anxiety score (lower or higher based on a median split) between subjects ANOVA. As shown in Figure 3, the analysis revealed a main effect of PoTJ, *F* (1,135) = 8.661, *p* = 0.004, η^2^ = 0.062, such that those who judged time to be passing by at a slower speed reported a greater tendency to overeat during the pandemic (*M* = 5.86, *SD* = 1.37) than those who judged time to pass at the same or at a faster speed (*M* = 5.21, *SD* = 0.95). There was no main effect of State Anxiety, *F*(1,135) = 2.814*, p* = 0.096, η^2^ = 0.021.

## Discussion

Past research has suggested that time perception and eating behaviors may be linked. The current investigation extends this theoretical perspective by examining how different temporal processes predict different eating behaviors during COVID-19 lockdown. Consistent with previous findings, which suggest that the two temporal processes of interest, namely interval timing and time passage, are distinct and rely on different mechanisms (e.g., Droit-Volet and Wearden, 2015), we observed in the current study that they did not correlate. Interval timing reflects the internal processing of time information and has served as an index for the internal clock model's pacemaker pulsating rate. Time passage judgement reflects the subjective temporal experience in response to the interaction between internal and environmental factors. Based on this distinction, we anticipated them to contribute differently to eating behaviors.

### Passage of time judgment as a predictor for snacking and overeating

The distinct contributions by interval timing and passage of time judgment were highlighted when we observed that passage of time judgment and time production predicted changes in eating habits differently. Regarding the passage of time judgment, and consistent with previous studies (Rodin, 1975; Crockett et al., 2015), we observed that participants who reported the feeling of time passing by slowly also reported a greater tendency to snack as well as to overeat during the lockdown. One interpretation of this frequent snacking aligns with the theoretical perspective related to temporal discounting and reward. As shown in past research, those who judge a duration to be longer are also more responsive to immediate rewards than to delayed rewards (Kim and Zauberman, 2009; Baumann and Odum, 2012; Suo et al., 2014) and vice versa (Vicario and Felmingham, 2018). Frequent snacking in the current study therefore may reflect the same mechanisms whereby the time between meals were experienced as long and slow, prompting the need for immediate food rewards.

The connection between the passage of time and overeating may reflect a coping strategy in response to the boredom experienced during the pandemic. Due to the environmental sameness while in quarantine, past studies have demonstrated a strong correlation between passage of time judgment and boredom (e.g., Ogden, 2020). Furthermore, the experience of boredom may relate to reductions in dopamine levels, which subsequently limit its availability to the reward system. We speculate that the tendency to overconsume food during the pandemic might have been a way in which an individual could have elevated dopamine to satisfy the reward system. This speculation is consistent with past studies showing that maintenance of healthy eating habits can be challenged by boredom, leading to emotional eating (Rodin, 1975; Crockett et al., 2015) that include behaviors such as eating sooner, more frequently, or eating a larger portion, subsequently increasing the sense of reward. Conversely, when not bored, and there is no need to satisfy the reward system, one would wait to consume food at a later time.

### Time production performance moderates the effect between time passage judgement and snacking

In addition to the passage of time experience, time production performance was also observed to interact with passage of time judgment, and together they predicted snacking frequency. Specifically, we observed that the effect of slow time passage on snacking frequency was amplified in those individuals who produced shorter durations (i.e., those with faster internal clock speeds) than those who did not. Putting the results into context, if an individual plans to eat in an hour, that approximated hour would arrive sooner for someone a with a slower pulsating clock. As such, when coupled with the feeling of time passing slowly, and the need to satisfy the reward system, this could amplify the tendency to snack earlier, and over time, accrue a higher number of snacking sessions.

Despite the evidence that time production contributed to the frequency of snacking, our results demonstrated only anecdotal evidence that this temporal process influenced the amount of food consumed. This null finding though may be an important distinction on how interval timing and passage of time judgment contribute to different eating behaviors. Whereas the feeling of how fast time is passing appears to be involved in both snacking and overeating, time production may contribute specifically to the “when” to eat rather than to the “how much” to eat. Importantly, this distinction could help pave ways for time-based therapies that address specific aspects of eating disorders.

### Mental health and eating behaviors

In addition to interval timing and passage of time experience, we also examined the potential contribution of anxiety and depression on eating habits during the pandemic lockdown. While all mental health measures were anticipated to influence eating behaviors, we only observed a contribution from state anxiety on overeating but not from trait anxiety nor depression. A speculation is that at the time of testing, we were in the early stages of the pandemic, and it is possible that depression and anxiety level had not significantly changed and therefore did not effectively alter eating behaviors from before the pandemic. A future study could consider chronic depression or anxiety and examine how each might contribute to eating patterns.

### Temporal experience and other psychological factors

While our study primarily focused on time perception as a predictor of eating behaviors, it is possible that temporal experience may operate in conjunction with other psychological factors that were triggered or enhanced during the pandemic. Take for example, personality traits and emotional responses. Similar to how time perception predicts eating behaviors in the current study, personality traits and emotional responses have also been shown to affect food consumption and snacking behaviors during the pandemic (e.g., Coulthard et al., 2021; McAtamney et al., 2021). Moreover, it has also been observed that higher order psychological factors such as personality, resilience, and the inability to process emotions (alexithymia) influenced symptoms of depression and anxiety during COVID-19 lockdowns, subsequently contributing to emotional eating habits (Cecchetto et al., 2021; Osimo et al., 2021). Given these findings, we speculate the possibility of an interaction or shared mechanisms between psychological factors and time perception on eating behaviors. In one manner, the effect of time perception on eating disorders could be modulated by personality or by the emotional responses surrounding the pandemic. In terms of the internal clock model (Gibbon, 1977), personality traits and emotions could modulate the level of arousal (and therefore controls the speed of the pacemaker), or levels of attention, resulting in different temporal experiences (e.g., Lehockey et al., 2018). Subsequently, these temporal experiences could have an impact on eating behaviors as reported in the current study.

In addition, prior research has also documented the contributions of higher cognitive processes to eating disorders (Manasse et al., 2015). Time perception could be linked or manifested within these higher cognitive processes, and in turn could contribute to eating habits associated with certain personality traits or emotions. For example, decision making has been shown to be influenced by timing and time perception (Wittmann and Paulus, 2008), as well as by one's personality traits (Franken and Muris, 2005; Bayram and Aydemir, 2017) and emotional responses (Andrade and Ariely, 2009; Lerner et al., 2015). This could subsequently contribute to one's decision regarding the amount and frequency of food consumption. In addition to decision making, deficits in cognitive flexibility (i.e., the ability to adapt to new, changing, or unplanned events) are also risk or maintenance factors for eating disorders (Manasse et al., 2015) including binge eating practices (Wang et al., 2021), resulting in the difficulty to change one's maladaptive eating routines to healthier practices. Moreover, attention to detail promotes fixation on disorder-relevant rituals such as calorie counting and body checking (Lavender et al., 2016; Haynos et al., 2018). It is possible that one's sense of time might interact with these individual differences, perhaps as a moderator for time-dependent eating rituals or habits (e.g., individuals whose internal clock operates at a faster speed might perform a calorie or body check more frequently).

In sum, based on evidence from past studies, we have speculated here how other factors might contribute to the relationship between time perception and eating behaviors. In one manner, personality traits and cognitive flexibility could modulate the emotional response to the lockdown conditions, which further impacts the experience of time perception and decision-making related to eating habits. In another manner, time perception could serve as a moderator between the psychological factors and eating practices.

### Summary

The current findings strengthen the general perspective that eating behaviors are tied to an individual's timing and time perception. Furthermore, given that the interval timing and the passage of time judgment predict changes in eating behaviors better than mental health, at least in our study, this implies that temporal cognition could serve as a potential predictor for an onset of poor eating habits that may not be detectable by more traditional measures such as mental health. The current study is also hopeful to provide a new research avenue that examines time perception in connection with other psychological factors associated with eating habits. This research approach could help provide a better understanding of eating behaviors and disorders. Subsequently, this is hopeful to aid in the development of novel interventions or treatments that employ cognitive-behavioral therapy methods that promote temporal awareness as an alternative approach toward treating eating disorders.

### Limitations

The primary goal of the current study was to seek evidence that connects temporal cognition and eating behaviors. While our data suggest both interval timing and passage of time to predict snacking or overeating tendencies, alternatively, it is also possible that these relationships are simply correlational. For instance, the abrupt changes in routines (e.g., work, school, extracurricular activities) and the necessity to quarantine might have facilitated the feeling of slow passage of time while simultaneously restricting food accessibility (Ammar et al., 2020) causing people to resort to less healthy options. Relatedly, it is also possible that food consumption was used as a coping mechanism (Buckland et al., 2021) to help relieve the psychological distress related to the pandemic. Future investigations are needed to rule out these possibilities.

In addition, snacking frequency was intended as an index of unhealthy eating behavior in the current study. However, different interpretations could have been made by the participants when responding to this survey question. In one case, one could interpret snacking as a form of unhealthy eating. Alternatively, snacking could also be interpreted as a practice of healthier eating (i.e., snacking, rather than full meals, is considered healthier; Smith, 2011). As such, we interpret our results with caution and encourage more precise operational definition in future experiments.

The current study employed two common time perception tasks, namely PoTJ and time production, to index the subjective and objective temporal experience, respectively. It is important to note that PoTJ is typically used in the context of relatively longer intervals (e.g., hours, days, etc.), whereas time production is typically administered for shorter intervals (e.g., milliseconds and seconds range), rendering different durations assessed during the current study. Future research may consider equating the duration intervals to directly compare PoTJ and time production performance. Relatedly, it would also be informative to examine how different timescales might consistently, or differently, predict eating behaviors.

## Data availability statement

The original contributions presented in the study are included in the article/Supplementary material, further inquiries can be directed to the corresponding author/s.

## Ethics statement

The studies involving human participants were reviewed and approved by University of Arizona. The patients/participants provided their written informed consent to participate in this study.

## Author contributions

EI and SL conceived, designed the study, collected, and analyzed the data. JT performed additional statistical analyses. All authors contributed to the writing of the manuscript and approved the submitted version.

## Funding

The study was funded by the College of Science and Department of Psychology at University of Arizona.

## Conflict of interest

The authors declare that the research was conducted in the absence of any commercial or financial relationships that could be construed as a potential conflict of interest.

## Publisher's note

All claims expressed in this article are solely those of the authors and do not necessarily represent those of their affiliated organizations, or those of the publisher, the editors and the reviewers. Any product that may be evaluated in this article, or claim that may be made by its manufacturer, is not guaranteed or endorsed by the publisher.
